# Vitreous Humor Positive for DNA of Human Herpesvirus 7 in Eye With Ocular Toxoplasmosis

**DOI:** 10.7759/cureus.41237

**Published:** 2023-06-30

**Authors:** Taishi Miyase, Kiyofumi Mochizuki, Satoko Kokuzawa, Ikumi Shiraki, Kazuhiro Murata, Hirokazu Sakaguchi

**Affiliations:** 1 Ophthalmology, Gifu University, Gifu, JPN; 2 Ophthalmology, Ogaki Municipal Hospital, Ogaki, JPN

**Keywords:** retina surgery, uveitis, vitreous humor, multiplex pcr, hhv-7, toxoplasmosis gondii

## Abstract

The aim of this article was to report our findings in a case of infectious uveitis in which the DNAs of both *Toxoplasma gondii *and human herpesvirus 7 (HHV-7) were detected in the vitreous fluid. A 31-year-old Brazilian man was examined in our hospital with a one-month history of blurred vision (20/40) in the right eye. He had been diagnosed with ocular toxoplasmosis of the right eye at nine years of age and has had repeated relapses. Because of the persistent vitreous opacities and refractoriness to acetylspiramycin and betamethasone, pars plana vitrectomy was performed. Multiplex PCR of the vitreous sample demonstrated the DNAs for both *T. gondii* and HHV-7. Trimethoprim/sulfamethoxazole with prednisone was prescribed. Six months after the beginning of the therapy, a resolution of the retinochoroiditis was found and the vision recovered to 20/25. Two months later, we performed a pars plana vitrectomy for an epiretinal membrane. The DNAs of both *T. gondii* and HHV-7 were not detected in the vitreous fluid and the epiretinal membrane. After continued treatment, the best-corrected visual acuity (BCVA) in the right eye improved to 20/16 and the metamorphopsia was reduced. It is inferred from this work that HHV-7 reactivation can activate refractory infectious uveitis in patients with chronic ocular toxoplasmosis.

## Introduction

Ocular toxoplasmosis is a common cause of posterior uveitis, and it is caused by an infection of the eye by *Toxoplasma gondii*, an obligate intracellular protozoan. Human herpesvirus 7 (HHV-7) infections have been associated with roseola infantum, acute hemiplegia of childhood, respiratory tract infections, and hepatitis. It has been reported that the DNA of *T. gondii* can be detected in the vitreous humor [1], and the DNA of HHV-7 can be detected in the tear fluid of patients before and after cataract surgery or after penetrating keratoplasty [2] and in the aqueous humor of patient with corneal endotheliitis [3]. However, to the best of our knowledge, the DNA of HHV-7 has not been detected in the vitreous.

We report our findings in a case of infectious uveitis in which the DNAs of both *T. gondii* and HHV-7 were detected in the vitreous sample collected during vitrectomy. 

## Case presentation

A 31-year-old Brazilian man was examined in our hospital, and he reported a one-month history of reduced vision in his right eye. He had a history of oral steroid treatment on multiple occasions since his diagnosis of ocular toxoplasmosis at nine years old.

Our ophthalmologic examination showed that his best-corrected visual acuity (BCVA) was 20/40 in the right eye and 20/13 in the left eye, and the intraocular pressure was 27 mmHg in the right eye and 17 mmHg in the left eye. Slit-lamp examination revealed corneal epithelial edema with anterior chamber inflammation in the right eye. The flare in the anterior chamber was a mild grade 1 flare in the SUN classification. 

**Figure 1:**
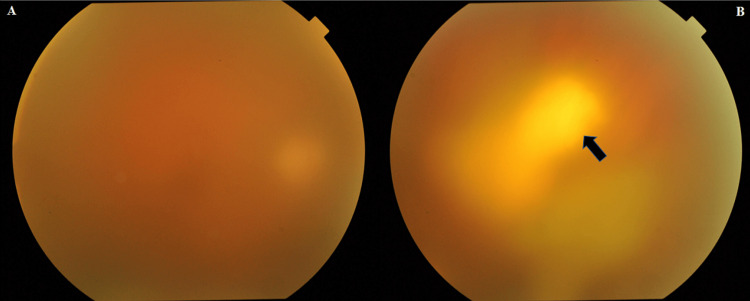
(A) Vitreous haze and (B) a yellowish-white lesion in the superior nasal region (arrow)

A funduscopic examination of the right eye showed a yellowish-white lesion in the superior nasal region with an overlying vitreous haze (Figure 1A, 1B). The examination findings of the left eye were unremarkable. All imaging studies and blood tests, including those for infection and autoimmune diseases, were negative except for elevated serum IgG antibody titers for *T. gondii*. Based on the ocular signs and a history of previous medications, he was diagnosed with recurrent ocular toxoplasmosis. 

Because the pre-diagnosis dosage of acetylspiramycin did not appear to be effective, the dose was increased to 1200 mg, and the oral corticosteroid was changed from prednisone to 2 mg of betamethasone [4,5]. One month later, the intraocular pressure had decreased, but the fundus findings had not improved. Because of the persistent vitreous opacities and low-amplitude electroretinograms, pars plana vitrectomy was performed after obtaining informed consent to determine a more accurate diagnosis.

**Figure 2:**
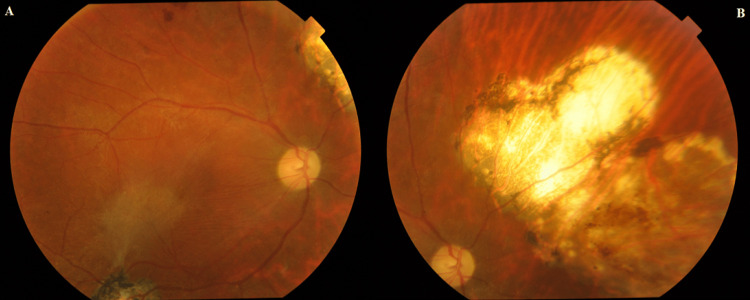
(A) A resolution of the vitreous opacities and (B) the areas of a yellowish-white mass lesion had improved with evidence of scar formation

**Figure 3:**
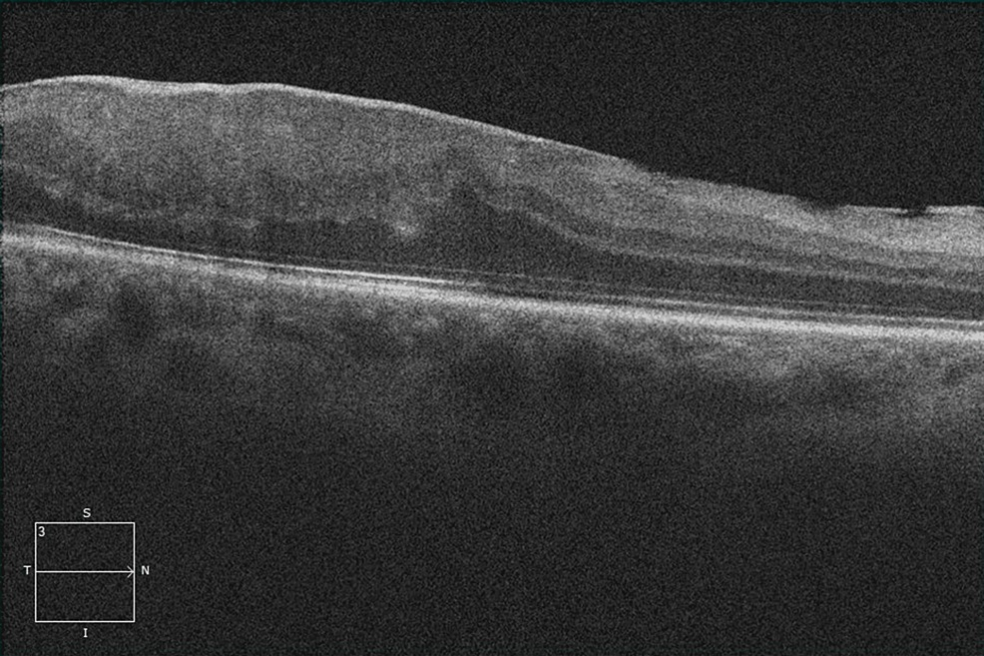
Optical coherence tomography revealed an epiretinal membrane in the right eye

An aliquot of vitreous humor was collected during the vitrectomy. Multiplex polymerase chain reaction (PCR) demonstrated the DNAs of both *T. gondii* (3.66×10^3^ copies/mL) and HHV-7 (4.77×10^3^ copies/mL) in the sample, but not herpes simplex virus types 1 or 2, varicella zoster virus, Epstein-Barr virus, cytomegalovirus, HHV-6, or HHV-8. Considering the possibility of immunodeficiency, thorough systemic examinations, including serologic testing for the human immunodeficiency virus (HIV), were performed, and all were negative. Although the same medications were continued after the surgery, the retinochoroiditis with overlying vitreous haze did not improve. After consultation with an infectious disease specialist, the patient began a regimen of trimethoprim/sulfamethoxazole (TMP/SMX; 10 mg/kg trimethoprim) with prednisone. Six months after beginning this therapy, the BCVA in the right eye improved to 20/25. Fundus examination revealed a resolution of the vitreous opacities, and the areas of a yellowish-white mass lesion had improved with evidence of scar formation (Figure 2A, 2B). However, optical coherence tomography revealed an epiretinal membrane in the right eye (Figure 3). 

Two months later, the patient underwent pars plana vitrectomy for the epiretinal membrane. The DNAs of both* T. gondii *and HHV-7 were not detected in the vitreous fluid or epiretinal membrane. One year after this surgery, the BCVA in the right eye improved to 20/16 and the metamorphopsia had decreased. There have been no recurrences despite a reduction in the corticosteroid and TMP/SMX dosages. 

## Discussion

Previous studies on HHV-7

To the best of our knowledge, this is the first report of a case in which the DNA of HHV-7 was detected in the vitreous fluid. There are some earlier studies that used multiplex PCR to search for HHV-7 in the ocular fluids, including the vitreous, of patients with ocular inflammation, and the DNA of HHV-7 was not detected in any of the cases [6-8]. On the other hand, Inoue et al. reported on patients with corneal endotheliitis in which genomic DNA for HHV-7 was detected by PCR analysis in the aqueous humor samples [3]. In addition, Shimomura used multiplex real-time PCR and detected the DNA of HHV-7 in the tear fluid before and after phacoemulsification and aspiration cataract surgery and after penetrating keratoplasty [2]. There have not been any reports of HHV-7 being detected in the vitreous fluid except for our case. Currently, neither a prophylactic vaccine nor a specific therapeutic agent has been developed to treat HHV-7 infections which are classified as beta-herpesvirus. HHV-7 has a phosphotransferase, and the possibility that foscarnet and cidofovir may be effective in its treatment has been reported. However, the possibility of strong side effects has also been discussed [9,10].

The pathogenicity of HHV-7

Primary infection by HHV-7 occurs mainly in early childhood, and the virus has been isolated through reactivation from latency in CD4+ T cells purified from the peripheral blood mononuclear cells of healthy individuals [11]. More than 90% of the general adult population has antibodies to HHV-7, but the pathogenicity of HHV-7 is less clear [12]. It was reported that reactivation of HHV-7 in individuals with neurological disease can occur if the patient is immunocompromised or HIV-infected [12,13]. 

The current case

Our patient with prolonged endophthalmitis had been treated with steroids multiple times, and he was detected with the DNAs of both *T. gondii* and HHV-7 by multiplex PCR of the vitreous humor, and the serum HIV was negative.

HHV-7 may have been reactivated by the immunosuppressive effects of the long-term use of steroid medications which impaired the naïve CD4 cells [14] and/or prolonged the intraocular inflammation associated with the ocular toxoplasmosis. In cases of persistent endophthalmitis such as recurrent ocular toxoplasmosis, multiplex PCR testing may be useful for the diagnosis and the effectiveness of the treatment. We have not used antiviral drugs such as foscarnet or cidofovir in this case, but we are considering administering them in case of a future recurrence.

Limitation

There is a limitation to this report. Serological testing for HHV-7 DNA in the blood circulation was not available in our hospital, and HHV-7 DNA in the blood was not performed. 

## Conclusions

We found that the DNAs of both *T. gondii* and HHV-7 were present in the vitreous fluid in the case of infectious uveitis. This is the first report of a case of HHV-7 being detected in the vitreous fluid. HHV-7 coinfection should be considered in cases of recurrent ocular toxoplasmosis.
